# The Effect of Self-care Educational/Training Interventions on the Outcomes of Gestational Diabetes: A Review Article

**Published:** 2018-12

**Authors:** Mitra KOLIVAND, Mehr Ali RAHIMI, Mohammad SHARIATI, Afsaneh KERAMAT, Mohammad Hassan EMAMIAN

**Affiliations:** 1. Student Research Committee, School of Nursing and Midwifery, Shahroud University of Medical Sciences, Shahroud, Iran; 2. Dept. of Reproductive Health, School of Nursing and Midwifery, Kermanshah University of Medical Sciences, Kermanshah, Iran; 3. Dept. of Internal Medicine, School of Medicine, Kermanshah University of Medical Sciences, Kermanshah, Iran; 4. Dept. of Community Medicine, School of Medicine, Tehran University of Medical Sciences, Tehran, Iran; 5. Reproductive Studies and Women’s Health Research Center, Shahroud University of Medical Sciences, Shahroud, Iran; 6. Center for Health Related Social and Behavioral Sciences Research, Shahroud University of Medical Sciences, Shahroud, Iran

**Keywords:** Gestational diabetes mellitus, Self-care, Education, Training

## Abstract

**Background:**

This study aimed to evaluate the effect of self-care educational/training interventions on gestational diabetes.

**Methods:**

In this review, we searched the ERIC, Clinical Key, Cochrane, Scopus, PubMed, and ISI databases as well as Iranian databases from 1990 until Jan 2017. Having evaluated 3267 articles by three of the authors, 20 clinical trials with educational/training approach remained for analysis. In this study, CONSORT checklist, JADAD scale and Cochrane handbook were used to evaluate the validity of articles.

**Results:**

The quality of 34% of articles was found to be poor due to probability of bias in designing the interventions and the effect of absence of blinding of personnel and participants. However, absence of blinding had a low impact on the results of most studies carried out on objective scales like blood glucose levels, or maternal and neonatal results. Moreover, 66% of studies were assessed to be at a good level of quality.

**Conclusion:**

There are few articles with educational/training approach on self-care in gestational diabetes mellitus, but based on the homogeneity of participants and significant results of self-care interventions, especially lifestyle ones, self-care guidelines can be developed for gestational diabetes mellitus.

## Introduction

Gestational diabetes mellitus (GDM) is a kind of diabetes that is first identified in pregnancy with an increase in blood glucose level (1). GDM is accompanied by maternal and neonatal complications, also usually disappears after birth, but the mother and child are prone to develop type II diabetes for the rest of their lives. The prevalence of GDM is on the rise, followed by increased type II diabetes (2,3). Using new criteria in an international multi-center study, 18% of pregnancies were accompanied by GDM (3). An incidence of 3.4% for GDM was reported in Iran (maximum and minimum levels to be 18.6 and 1.3%, respectively), which is in line with the report in Kermanshah, Iran as well as the results of the systematic review (4–6).

Although the increased incidence of GDM has caused serious concerns for the health system around the world (7), there is strong evidence suggesting that a proper management can yield better results for the mother and child (8). Hence, women should receive education on diabetes self-care according to national standards after diagnosis and based on their requirements (9). The self-care approach was evaluated to diabetes changes during 1970–1990 from the patient’s passive participatory approach to patient’s empowerment and active participation in management of their disease (10), an approach we expect to occur in providing healthcare to mothers with GDM. With regard to GDM increase rate, few studies have been performed on each domains of self-care in gestational diabetes. The need for a new insight into the necessity of self-care educational program for GDM along with the current approach of the international community made the researchers in this study to carry out a systematic review to evaluate the effect of self-care educational/training interventions on GDM.

## Methods

The research question in this study was: What is the effect of self-care education interventions on pregnancy outcomes in women with gestational diabetes versus routine training and services?

### Strategy of search

Educational interventions included those performed with educational, teaching, instructional, training or counseling methods in the self-care domains of GDM. At baseline, using mesh and keywords of several articles, the keywords compatible with the research objectives in English and Persian. The keywords were searched in the educational resources information center (ERIC), Clinical Key, Cochrane, Scopus, PubMed, and ISI databases as well as Iranian databases, including Magiran, SID and Iran Medex until Jan 2017. Advanced search was carried out in the PubMed database using the operators AND/OR, keywords derived from the MESH and studies related, and limitations of the clinical trial articles without time limitations. With finding more relevant keywords, such as “self-care education”, “self- care education /training” and “gestational diabetes”, search done in other databases (English and Persian).

Keywords were gestational diabetes mellitus, diabetes pregnancy, self-care, self-management, education, training.

### Selection of studies

The articles were screened by their titles and abstracts. The studies conducted on the women diagnosed with GDM were selected for analysis. The inclusion criteria consisted of articles with a relevant subject, a randomized clinical trial method, a full text and written in English or Persian language, determining the research question with PICOS model, the precise design of a randomized controlled clinical trial, without limitation of print time, paper or dissertation approved in academic centers. The educational interventions on self-care by every member of the healthcare team, by every educational/training method, in group or individuals were included in the study. Further, the studies with interventions approved by the ethical committee of research were selected for analysis.

For grey literature, Persian dissertations and unpublished researches through the National Library of the Islamic Republic of Iran and the Iranian Institute of Science and Technology (Iran Doc) and library resources of the universities as well as the WHO and Iranian Center of Clinical Trials Registration were searched. For English dissertations and unpublished researches we searched various sources, including the dissertation.com site, as well as the WHO and US Center of Clinical Trials Registration.

All stages of assessment of the quality of articles were carried out by two independent researchers. In the case of disagreement between the reviewers, a third reviewer refereed the articles.

### Validity assessment of articles

CONSORT checklist (11), JADAD scale (12,13) and Cochrane handbook (14) were used to evaluate the validity of articles. CONSORT checklist was first used to assess the quality of the selected papers, out of which only two articles were found to have less than half of the items. JADAD scale is scored from 0 to 5 according to the probability of bias in the sampling process, sample loss and correct blinding. Studies with a score of ≥3 have a good quality (12,13). In addition, different kinds of bias defined in Cochrane handbook were used to assess the internal validity of articles (14). The external validity of the articles was also analyzed, and the papers with participants representing the target population as well as the samples randomly accessible from the population were selected for analysis. In most of the studies, the participants included those referring to the clinics and university hospitals from others prenatal clinics or offices.

### Risk of bias

In the selection bias, the random sequence generation item was evaluated to be at low risk due to the use of one random replacement method in all studies. As for allocation concealment, 14 articles were assessed to be unknown and 6 articles were evaluated to be at low risk. In examining the performance bias, the blinding of participants and personnel was found to be unknown in 15 articles and at low risk in 5 articles. As for detection bias, all cases were found to be at low risk. In attrition bias, 4 papers were reported to be at high risk and 16 papers were at low risk. In reporting bias, due to the presence of all primary and secondary results in the report, all papers were evaluated to be at low risk. (Table 1). The quality of 35% of articles was found to be poor due to probability of bias in designing the interventions and the effect of absence of blinding of personnel and participants. Bias was possible to occur in the studies using self-reporting scales with no blinding of participants. However, absence of blinding had a low impact on the results in most studies carried out on objective scales like blood glucose levels or maternal and neonatal results, and the rest of studies were assessed to be at a good level of quality.

**Table 1:** Cochrane bias risk assessment for included studies

***Reference***	***Random sequence generation***	***Allocation concealment***	***Blinding of participants and personnel***	***Blinding of Incomplete outcome assessment***	***outcome data***	***Selective reporting***	***Other sourcesof bias***
Mendelson et al 2008	L	U	U	L	L	L	L
Cao et al 2012	L	U	U	L	L	L	L
Youngwanichsetha et al 2014	L	L	U	L	L	L	L
Abirami et al 2014	L	U	U	L	L	L	L
Petkova et al 2011	L	U	U	L	H	L	H
Viswanath et al 2014	L	U	U	L	H	L	H
Amason 2013	L	U	U	L	L	L	H
Valipur et al 2014	L	U	L	L	L	L	L
Reader et al 2006	L	U	U	L	L	L	L
Hasle et al 2015	L	L	U	L	L	L	L
De Barros et al 2010	L	L	L	L	L	L	L
Brankston et al 2004	L	L	U	L	L	L	H
Avery et al 1997	L	U	U	L	L	L	H
Ferrara et al 2011	L	U	L	L	L	L	H
Yang et al 2014	L	U	L	L	H	L	H
Bo et al 2014)	L	L	L	L	L	L	L
Kaviani et al 2014	L	U	U	L	L	L	L
Khadivzadeh et al 2015	L	L	U	L	L	L	L
Kaveh et al 2012	L	U	U	L	H	L	L
Homko 2002	L	U	U	L	L	L	L

L= Low Risk H= High Risk U= Unclear

## Results

**Fig. 1:**
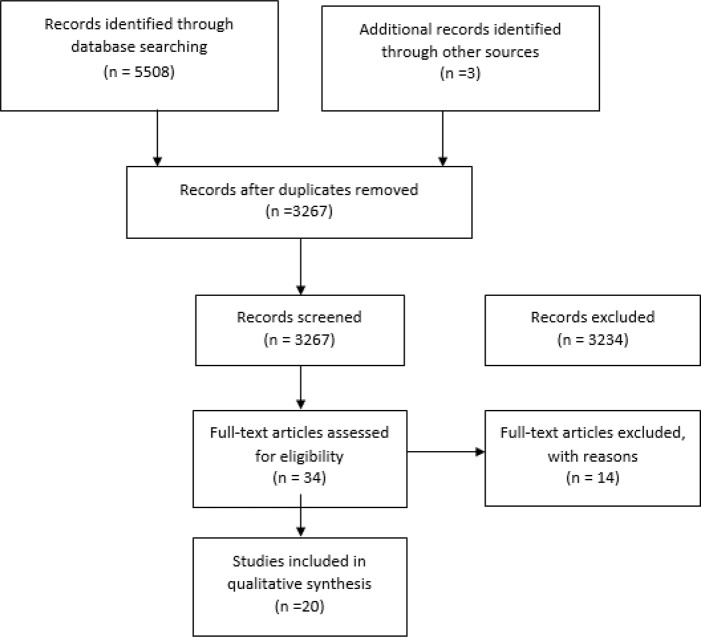
Flow diagram of the literature review process (PRISMA)

From 5508 articles found, 3267 articles remind after excluding the repetitive ones. In the search for the theses, two English and one Persian thesis were found. The titles and abstracts of the articles were then studied and those irrelevant to the research objectives were excluded and the full text of the relevant and probably relevant articles along with their references were evaluated to include other possible sources in the analysis. The articles lacking the full text (10 articles) were excluded due to the absence of adequate information in the abstract. After excluding the abstracts, non-clinical trials and studies irrelevant to the self-care educational/training interventions (32 articles), 34 articles remained. Of these, the clinical trials without randomization were excluded from the study (14 articles). Finally, 20 articles with educational/training approach remained three in Persian and the rest in English language. The algorithm of analysis is indicated in Fig. 1.

The outcomes under analysis comprised of knowledge level, self-care behaviors, glycemic control, neonatal, maternal, and obstetric results.

### Characteristics of studies

Table 2 illustrates the features of the selected papers. This systematic review included 20 studies on 2698 women with GDM (1354 in intervention group and 1344 in control group). The intervention providers in these studies comprised of nurses, nutritionists and counselors, pharmacologists, doctors, midwives, and sports physiologists. Interventions were performed on different domains of knowledge, lifestyle modification, nutritional modification, physical activity, relaxation and blood glucose self-monitoring.

**Table 2:** Characteristics and key results of included studies

***First author & year***	***Intervention type***	***Participants IG (intervention group) CG (control group)***	***Target population***	***Gestational week at start***	***results***
**Self- care education Interventions**					
Khadivzadeh, T. 2015	Education of self-care	30 GDM women in each group	GDM^*^ women referred to Ommolbanin hospital- Mashhad-IRAN	24–30	- Nutritional compliance (*P*<0.001) - Physical activity adherence (*P*<0.001) - SMBG^*^ adherence (*P*<0.001) - Drug adherence (*P*<0.001) - Total self- care score (*P*<0.001) - Perceived stress score (*P*=0.02)
Viswanath, L 2014	Teaching on self-care, monitoring of dietary log, guidance for diet planning and lifestyle change	20 GDM women in each group	GDM women attending the Obstetrical unit of Amrita Institute of Medical Sciences, Kochi- Kerala-INDIA	24–28	- Increase of self-care agency (*P*<0.001)
**Informational Interventions**					
Amason, J.S. 2013	Education for knowledge increase	18 in IG 5 in CG	GDM women at the Obstetric /Gynecology offices in the southeastern United States	32–36	- Knowledge of diabetes with cohen test (*P*<0.5) - Gestational weeks at delivery (*P*=.005)
Mendelson, SH.G. 2008	A supplementary education session	49 in IG 51 in CG	GDM women referred by their obstetric provider to a hospital-based Perinatology clinic- Los Angeles, USA	12–32	- Health Promoting Lifestyle Profile II scores (*P*<0.016) - Stress management (p=0.003) - Health Responsibility (*P*=0.000) - Nutrition (*P*=0.001) - Physical activity (*P*=0.000) - Spiritual Growth (*P*=0.005)
Petkova, V. 2011	An education program	15 in each group	GDM women attending antenatal clinic - Sofia- Bulgaria	From 28 in diagnose	- Knowledge level (*P*<0.001) - Changes in quality of life items: - Happy and in good mood (*P*=0.021) - Calm (*P*=0.001) - Vital and active (*P*<0.001) - Woke up fresh and rested (*P*<0.001) - Daily routine full with - Interesting things (*P*<0.001)
Kaveh, M. 2012	Instruct education	31 in IG 30 in CG	GDM women, referred to hospitals of Shiraz University of Medical Sciences-IRAN	21–34	- Knowledge grade (*P*<0.001) - 1 hpp*, 2hpp (*P*<0.001, *P*<0.001)
**Life Style Interventions**					
Cao, XP. 2012	Individualized diabetes education, dietary and exercise advice, and instructions to SMBG^*^	127 in IG 148 in CG	GDM women from the outpatient clinic at the First Affiliated Hospital of Sun Yat-sen University (Guangzhou, China)	IG from 30.04± 4.46 CG from 30.82 ± 4.91	Lower instances of: - Premature delivery (*P*<0.05) - Neonatal care unit admission (*P*<0.05) - Neonatal birth weight (*P*<0.0001) - Waist circumference (*P*<0.01) - Lower 30-min glucose levels after a 75-g glucose load (*P*<0.05) - High-density lipoprotein levels (*P*<0.05)
Youngwanichsetha S. 2014	Training of Yoga exercise & mindfulness eating	87 in IG 90 in CG	GDM women referred to a tertiary hospital in southern Thailand (the referral center for diabetes care)	24–30	- FBS (*P*<0.012) - 2hpp (*P*<0.001) - Glycosylated hemoglobin (HbA1c) (*P*<0.016)
Abirami. P. 2014	Multi Model Intervention: counseling about diet, exercise, SMBG, insulin therapy and yoga	104 in IG 108 in CG	GDM women attended the antenatal outpatient department at government hospital, Tambaram- INDIA	24–28	- FBS in 28,32 & 36 (*P*=0.001, *P*=0.001, *P*=0.001) - 2 hpp (*P*=0.001, *P*=0.001, *P*=0.001)
Ferrara, A. 2011	Diet, Exercise and Breast feeding Intervention (DEBI)	96 in IG 101 in CG	GDM women attending to the Kaiser Permanente Medical Care Program of Northern California- USA	After diagnosis in IG 31.8 (5.6) & in CG 31.0 (6.1)	- Dietary fat intake (*P*=0.002)
Yang, X. 2014	Life style intervention	343 in IG 362 in CG	Women with plasma glucose (PG) at GCT ≥7.8 mmol/L were referred to TWCHC GDM Clinic-Tiangin, CHINA	After screening in 24–28	- Weight gain from entry to 34th gestational week (*P*<0.012) - Neonatal birth weight (*P*=0.021) - Apgar score <7 in first minute (*P*<0.016) - Preeclampsia in IG (*P*<0.031)
Bo, S. 2014	Exercise and behavioral recommendations and counseling	200 participants (Undetermine in each group)	GDM women attending the Sant’Anna Hospital-Turin, ITALY	After diagnosis in 24–26	- 2 hpp (*P*<0.001) - HbA_1_C (*P*<0.001) - Log-triglycerides (*P*=0.02) - Maternal/neonatal complications (*P*=0.02)
**Exercise Training Interventions**					
Hasle, RH.E. 2015	Exercise training with stationary ergometer	20 in each group	GDM women were recruited from the Diabetes Service at King Edward Memorial Hospital, Perth, Western Australia	26–30	- Improved maternal fitness in two groups (*P*<0.05) - Improved aerobic fitness in IG (*P*<0.001) - Improved of exercise attitude (*P*<0.001) - Improved intention to exercise (*P*<0.05)
De Barros, M.C. 2010	Training of resistance exercise program	32 in each group	GDM women who were under prenatal follow-up at the Obstetric Clinic of the University Hospital, University of Sao Paulo School of Medicine – Brazil	24–34	- Number of patients need to insulin (*P*<0.005) - Time spent within the target glucose range (*P*<0.006)
Brankston, G.N. 2004	Circuit-type resistance training	16 in each group	GDM women cared for at one of two perinatal units in Edmonton, Alberta, Canada	26–32	- Amount of insulin required (*P*<0.05) - Latency to insulin requirement (*P*<0.05) - Pooled post meal Blood glucose (*P*<0.05)
Avery, M.D. 1997	Home based aerobic exercise training with cycle Ergometer and walking	15 in IG 14 in CG	GDM women referred to a large Midwestern health maintenance organization- Minnesota university-USA	≥32	- Cardiorespiratory fitness (*P*<0.005) - Daily carbohydrate consumption in CG (*P*<0.03) - Increase in exercise, leisure & total activity in IG (*P*=0.01, *P*=0.03, *P*=0.002)
**Nutritional education Interventions**					
Valipur. GH. 2014	DASH^*^ Diet education	16 in each group	GDM women attending in fertility clinics of medical university of Kashan-IRAN	24–28	- FBS (*P*<0.02) - Insulin level of serum (*P*<0.03) - Intake of carbohydrate, oil, fiber, simple glucose, SFA, PUFA, fruit, vegetable, nuts, minerals & micronutrient (*P*<0.0001) - Intake of cholesterol (*P*<0.0001) - HOMA-IR score (*P*=0.03) - Total antioxidant capacity (*P*<0.0001) - Total glutathione levels (*P*<0.0001)
Reader, D. 2006	Nutrition practice guidelines education	85 in routine dietetic care & 130 in new guideline	GDM women attending in clinics (diabetes clinics, obstetric and other clinic types)- USA	After diagnosis of GDM	- No significant differences
**Mental Health Interventions**					
Kaviani, M. 2014	Benson’s relaxation technique	29 in each group	GDM women referred to Hafez medical university of Shiraz- IRAN	24–30	- FBS (*P*<0.001) - 2 hpp (*P*<0.001) - Systolic Blood pressure (*P*<0.006)
Khadivzadeh, T. 2015	Self-care education	30 in each group	GDM women referred to Omolbanin hospital-Mashhad- IRAN	24–30	- perceived stress score (*P*=0.02) - Diet adherence (*P*<0.001) - Physical activity adherence (*P*<0.001) - Self monitoring of Blood glucose (*P*=0.001) - Drug adherence (*P*=0.01) - Total self-care score (*P*<0.001)
**Educational Interventions of Blood glucose self-Monitoring**					
Homko, C.A. 2002	Self-monitoring of Blood glucose	31 in IG 27 in CG	GDM women participate in the Diabetes-in-Pregnancy Program at Temple University Hospital and/or 1 of its satellite hospitals- USA	≥33	- No significant differences

### Self-care education

Only one study was found to have complete educational content about self-care education (15). The effects of this intervention on self-efficacy and self-care behaviors were observed in these studies (15,16). Assessment of the impact of self-care training, a significant difference was found in the total score of self-care and its domains consist of adher to diet, drug use, physical activity, and blood glucose self-monitoring (15). Moreover, an increased score showed for self-care and its three dimensions (16).

### Knowledge development

Although most of the interventions presented a kind of education in different domains of self-care, few studies had been done to enhance the participant’s knowledge (17–20). Several interventions were found to have improved the knowledge level (17,19, 20) or health promotion behaviors (18). Interventions were performed over one (17,18) or three training sessions (19,20). The maternal or neonatal outcomes, except for preterm birth (17), showed no significant difference between groups (17,18).

### Lifestyle education

The maximum number of articles obtained was related to lifestyle interventions, a total of 7 articles being included in the study (21–26). Nutritional education and sports training were the basis of these articles. Glycemic control with a significant reduction of fast blood sugar (FBS) (22,23), blood glucose two hours postprandial (22–25) and hemoglobin A1c (HbA1c) (22,26) was seen in these interventions. In a study, lack of significant difference of FBS (26) and abnormal HbA1c was reported (25). Lack of difference in maternal and neonatal outcomes (21,25, 26) were some of the findings of these studies.

### Exercise interventions

In this study, the exercise interventions with educational/training approach were included (27–30). In exercise interventions, aerobic fitness improvement, exercise attitude, exercise intention and cardiovascular fitness (27,30), as well as contradictory results, were observed with regard to the effect of resistance exercise on the number of patients requiring insulin and prescribed insulin dose (28,29). These interventions did not have a significant impact on glycemic control (28,29).

### Nutritional interventions

The majority of interventional studies in this domain compared various diets in GDM, and only three studies were found to have educational approach (18,31, 32). The nutrition education intervention with dietary approaches to stop hypertension (DASH) performed significantly reduced FBS and serum insulin level of blood (31). However, teaching the practical guidelines of nutrition, compared with conventional nutrition training and diets, showed no significant difference in glycemic control or other maternal or neonatal outcomes (32).

### Mental health interventions

No study was found with exactly this title in the searches. Relaxation training through Benson method in the study improved glycemic control and systolic blood pressure (33). The self-care education intervention carried out significantly reduced perceived stress by proposing problem troubleshooting, using problem-solving skills and group discussion strategies and presenting the experiences of other mothers in coping with their disease and problems (15).

### Blood glucose self-monitoring

The studies on blood glucose measurement mostly concentrated on three domains of measurement times, comparison of self-monitoring with regular blood glucose monitoring system and use of telemedicine. The only educational/training intervention assessing the effect of blood glucose self-monitoring on self-efficacy and outcomes of pregnancy in GDM was conducted (34), revealing no significant difference between groups in blood glucose control.

## Discussion

Numerous studies have been carried out around the world on self-care in type I and II diabetes, but the position of this approach is still not recognized in the management of GDM. Improvement of the self-care behaviors reported in a limited number of studies (15,16, 35) is indicative of the efficacy of and necessity of these interventions. The importance of self-care education and training in GDM not only helps the management of disease and reduction of maternal and neonatal complications but also will reduce the risk of maternal and neonatal type II diabetes in the upcoming years in the case of persistent changes in the lifestyle of people.

Increasing the knowledge level is one of the basic foundations of every self-care educational/training program. Evidently, this stage should be more taken into account in less developed societies.

In lifestyle studies, the interventions compatible with national cultures such as use of yoga or approaches like mindfulness eating (22,23, 25) in Asian countries. This approach recommended by international organization (36,37).

In exercise interventions, common characteristics, and to some extent different from other interventions, were seen, including low number of participants, considering fetal health approval in addition to maternal health in inclusion criteria, considering all known principles of exercise in pregnancy, supervised sessions and necessity of blood glucose measurement before starting exercise (27–30). The insignificant difference in obstetric results or glycemic control in these studies (38–41) might be due to small sample size. The mechanism of exercise effect on GDM was similar to type II diabetes and stated that exercise probably increased sensitivity to insulin (42). There was more tendency to use physical activities and exercise simultaneously to manage GDM over the past few years.

As for the educational interventions studied, a significant increase in nutritional knowledge, improved FBS, serum insulin level, glycemic control and quality of life were observed (19,25). Most of the available nutritional guidelines were on types I and II diabetes, and no specific instructions were designed for GDM (32). Studies on different diets in GDM are being conducted, and no definite results have been obtained so far.

In fact, reducing stress and improving the psychological status of individuals can be the incidental effects of every education and training. The results of the limited existing studies (15,20) were the same as those of other studies (35,43). Reducing stress and promoting the mental health were the positive effects of these interventions, which could favorably enhance glycemic control by decreasing the stress hormones and promoting the sense of self-efficacy ( 15,20,35,43). Mental health care is necessary for the mothers diagnosed with GDM.

In spite to result of another study (34), if we accept the blood glucose self-monitoring includes two sections of test performance and blood glucose model management, its position and role in the management of disease will be established.

## Conclusion

There are few articles with educational/training or counseling approach to self-care in GDM than in type I and II diabetes. The existing studies have been carried out on various populations around the world, with different approaches, at various centers with limited number of samples. Despite these limitations and difficulty of interventional studies on pregnancy, their common findings can be acceptably generalized considering the homogeneity of participants, including gender, age (reproductive age), gestational diabetes, lack of other important diseases and referral to diabetes clinics of university centers. Thus, based on the homogeneity of participants and effective results of self-care interventions, especially lifestyle ones, self-care guide and guidelines can be developed for GDM. Although low number of studies or samples in some self-care domains were some limitations of this study, this study was the first to assess the need for self-care education in GDM.

## Ethical considerations

Ethical issues (Including plagiarism, informed consent, misconduct, data fabrication and/or falsification, double publication and/or submission, redundancy, etc.) have been completely observed by the authors.
